# Radiotherapy of the Hepatocellular Carcinoma in Mice Has a Time-Of-Day-Dependent Impact on the Mouse Hippocampus

**DOI:** 10.3390/cells12010061

**Published:** 2022-12-23

**Authors:** Mona Yassine, Soha A. Hassan, Simon Sommer, Lea Aylin Yücel, Hanna Bellert, Johanna Hallenberger, Dennis Sohn, Horst-Werner Korf, Charlotte von Gall, Amira A. H. Ali

**Affiliations:** 1 Institute of Anatomy II, Medical Faculty, Heinrich-Heine-University, Moorenstrasse 5, 40225 Düsseldorf, Germany; 2 Zoology Department, Faculty of Science, Suez University, Cairo-Suez Road, Suez 43533, Egypt; 3 Laboratory of Molecular Radiooncology, Clinic and Policlinic for Radiation Therapy and Radiooncology, Medical Faculty, Heinrich-Heine-University, Universität Strasse 1, 40225 Düsseldorf, Germany; 4 Institute of Anatomy I, Medical Faculty, Heinrich-Heine-University, Moorenstrasse 5, 40225 Düsseldorf, Germany; 5 Department of Human Anatomy and Embryology, Medical Faculty, Mansoura University, El-Gomhoria St. 1, Mansoura 35516, Egypt

**Keywords:** chronotherapy, circadian clock, clock genes, cytokines, hepatocellular carcinoma, hippocampus, neuroinflammation, p-ERK, phenobarbital, radiotherapy

## Abstract

Chronic liver diseases including hepatocellular carcinoma (HCC) create a state of chronic inflammation that affects the brain via the liver–brain axis leading to an alteration of neurotransmission and cognition. However, little is known about the effects of HCC on the hippocampus, the key brain region for learning and memory. Moreover, radiotherapy used to treat HCC has severe side effects that impair patients’ life quality. Thus, designing optimal strategies, such as chronotherapy, to enhance the efficacy and reduce the side effects of HCC treatment is critically important. We addressed the effects of HCC and the timed administration of radiotherapy in mice on the expression of pro-inflammatory cytokines, clock genes, markers for glial activation, oxidative stress, neuronal activity and proliferation in the hippocampal neurogenic niche. Our data showed that HCC induced the upregulation of genes encoding for pro-inflammatory cytokines, altered clock gene expressions and reduced proliferation in the hippocampus. Radiotherapy, in particular when applied during the light/inactive phase enhanced all these effects in addition to glial activation, increased oxidative stress, decreased neuronal activity and increased levels of phospho(p)-ERK. Our results suggested an interaction of the circadian molecular clockwork and the brain’s innate immune system as key players in liver–brain crosstalk in HCC and that radiotherapy when applied during the light/inactive phase induced the most profound alterations in the hippocampus.

## 1. Introduction

Hepatocellular carcinoma (HCC) represents the most prevalent primary hepatic malignancy and the third leading cause of cancer deaths with an 8.3% mortality rate [1]. Importantly, HCC is frequently discovered in an advanced stage due to the absence of specific symptoms and lack of diagnostic markers in the early stages [2]. Generally, anti-cancer treatment protocols have severe side effects, including cardiotoxicity, neurotoxicity, sexual dysfunction, infertility and fatigue that can dramatically impact patients’ quality of life [3]. Thus, designing proper management strategies and new therapeutic approaches are required for improving the efficacy of HCC treatment and reducing the severity of the side effects. 

HCC pathogenesis includes inflammatory damage of hepatocytes and regeneration leading to fibrotic deposition, distorted hepatic architecture and finally tumor development associated with liver function impairment [4]. Recently, several studies have focused on the liver–brain axis, which is mediated mainly via the autonomic nervous system including the vagus nerve [5]. Furthermore, chronic peripheral diseases including cancer [6] which create a state of chronic inflammation have been shown to impact the brain by means of microglial activation, secretion of pro-inflammatory cytokines such as IL-1b, IL-6 and TNF-α [7] and alterations in neurotransmission and behavior [8,9]. In particular, poor memory and impaired concentration as well as disturbances of circadian rhythms and sleep are common cancer-associated features. 

The circadian system in mammals is a hierarchically organized internal time-keeping system which coordinates rhythms of physiology and behavior with the 24h solar day [10]. The hypothalamic suprachiasmatic nucleus (SCN) receives time information and orchestrates subordinate clocks in the brain and the periphery [11]. Almost each cell contains intrinsic molecular clockwork, which is composed of autoregulatory transcriptional/translational feedback loops [11]. The core clock consists of the two positive transcription factors CLOCK and BMAL1 in addition to two negative regulators, the PERs (PER1 and PER2) and the CRYs (CRY1 and CRY2). An accessory feedback loop, including the orphan nuclear receptors REV-ERBa and RORα, provides further stabilization for the clock machinery [12]. The circadian system modulates drug metabolism and toxicity depending on the time of administration and therefore, some medications show a higher efficacy at a specific time-of-day [13]. Based on this, chronotherapy, a novel therapeutic approach depending on the timed delivery of treatments, has been suggested to reduce the toxicity and enhance the treatment outcomes [14,15]. Interestingly, chronotherapy has been implemented in anticancer drug administration and displayed promising outcomes in human patients, especially in metastatic colorectal cancer and glioblastoma [15]. Recently, we showed in a mouse model that radiotherapy applied in the late dark/activity phase had the highest anticancer efficacy on tumor tissue and the least side effects on the surrounding non-tumor liver tissue in HCC-bearing mice [16]. In contrast, radiotherapy applied in the early light/inactive phase showed the least effects on stress hormone levels, SCN neuronal activity and rhythmic locomotor activity [17]. These data indicate that radiotherapy applied in different phases has diverse (side) effects on the tumor, liver and SCN. However, little is known about the effects of HCC and timed radiotherapy on other brain regions.

In this study with a mouse model, we analyzed the effect of HCC and the timed application of radiotherapy on the expression of pro-inflammatory cytokines, clock genes, markers for glial activation, oxidative stress, neuronal activity and neural progenitor cell proliferation in the hippocampal neurogenic niche, a key structure for neural plasticity and cognition. Additionally, we investigated the protein levels of phosphorylated extracellular signal-regulated kinase 1/2 (ERK1/2), an essential modulator of hippocampal functions (Medina and Viola, 2018). Chronic treatment with phenobarbital (PB) that was used here to promote HCC development affected the expression of pro-inflammatory cytokines and clock genes as well as microglia activation in the hippocampus. HCC resulted in an upregulation of the expression of pro-inflammatory cytokines, a downregulation of neural progenitor cell proliferation and p-ERK protein levels as well as changes in clock gene expression in the hippocampus. Radiotherapy, in particular when applied during the light/inactive phase of mice, enhanced all of these effects and resulted in glia activation, increased oxidative stress and changes in neuronal activity in the hippocampus. 

## 2. Material and Methods 

### 2.1. Animals

Male mice (PER2::LUC with C57BL/6J background) were used [16,17]. Mice were kept in groups of 3–4 animals in standard cages with free access to water and food (*ad libitum*) in temperature-controlled rooms under normal light–dark cycles (12 h:12 h, lights on 06:00 am, Zeitgeber time (ZT)00, light off 06:00 pm, ZT12). Experiments during the dark phase were performed under dim red light.

Details on the treatment of the mice are described in [16]. Briefly, to induce HCC development, a cohort of mice aged 14 days were injected intraperitoneally (i.p.) with a single dose of diethyl nitrosamine (DEN) (10 mg/kg, Cat # N0756-10ML, Sigma Aldrich, St. Louis, MI, USA). To promote tumor development, 0.05% phenobarbital (Luminal, Cat # PZN-04895270, Desitin, Hamburg, Germany) was added to the drinking water for 7 months according to previous studies [18,19,20,21]. All mice survived tumor induction. Development of HCC was confirmed via postmortem inspection of the liver and magnetic-resonance imaging (MRI) [16]. A parallel cohort of mice was treated with PB for 7 months without DEN injection (Control-PB). Another group of mice that received neither PB nor DEN served as the untreated group. All animal experiments were performed at the Central Facility for Animal Research and Animal Welfare (ZETT) at Düsseldorf University, Germany. Animal experiments were approved by the Regional Council Darmstadt and the North Rhine-Westphalia State Agency for Nature, Environment and Consumer Protection (LANUV), Germany (Reference number: AZ 81-02.04.2018-A146) and conformed to federal guidelines and Directive 2010/63/EU of the European Union of Animal Care.

### 2.2. Irradiation

Details on the irradiation of the mice are described in [16]. Briefly, HCC-bearing animals at the age of 7–8 months were randomly divided into irradiation and non-irradiation groups. Irradiation was performed at ZT02, ZT08, ZT14 or ZT20 (*n* = 3 for each time point). Prior to irradiation, the mice were anesthetized by i.p. injection of a mixture of ketamine/xylazine (100/10 mg/kg) and then fixed with an adhesive tape so that their abdomen was facing the irradiation source. Mice were exposed to a dose of 10 Gray (Gy) for 10 min at (175 kV and 15 mA) using a Gulmay RS225 X-ray system (XStrahl Europe, Ratingen, Germany). A total of 10 Gy was used according to the dose applied in human palliative radiotherapy [22]. Mice were returned to their home cages and sacrificed forty-eight hours after the application of radiotherapy at ZT02, ZT08, ZT14 or ZT20, respectively. Mice in the control groups were handled in the same way at ZT02, ZT08, ZT14 or ZT20, but they did not receive radiotherapy. Per each group, 12 mice were used, *n* = 3 mice for each time point, to keep the scarification of all mice close to each other in every time point. Only animals with a similar tumor size were included for further analyses; animals without tumors or with smaller or larger tumors were excluded.

### 2.3. Real Time qPCR

Mice from each group were killed at four different time points: ZT02, ZT08, ZT14 and ZT20, 48 h after irradiation/handling. The entire hippocampus was dissected, snap frozen and then stored at −80 °C. RNA was isolated using RNeasy Plus Universal Mini Kit (Cat # 73404, Qiagen, Venlo, Netherlands) according to the manufacturer’s instructions. The concentration of RNA in the samples was analyzed using a NanoDrop 2000 spectrophotometer (Thermo Scientific, Massachusetts, USA) and its purity was assessed by the ratio of absorbance at 260 nm and 280 nm. A Revert Aid First Strand cDNA Synthesis Kit (Cat # K1621, Thermo Scientific, Waltham, MA, USA) was utilized to prepare cDNA using 1 µg RNA. Negative controls were used to exclude the DNA contamination. The expression levels of several genes encoding for pro-inflammatory cytokines, including *IL-1a*, *IL-1b*, *IL-1r1*, *IL-6* and *TNFα*, as well as the expression of clock genes, *Clock*, *Bmal1*, *Per1*, *Per2*, *Cry1*, *Cry2* and *Rev-Erba* were analyzed. The sequences of the used primers are listed in Table 1.

Real-time qPCR was performed using 1 ng cDNA and SYBR GREEN (Cat # KK4605, Kapa Abi-Prism, Sigma-Aldrich, St. Louis, MI, USA), in an ABI StepOne Plus Real-Time PCR System (Applied Biosystems, Waltham, MA, USA) with the following program: activation at 95 °C for 5 min, then denaturation with 40 cycles at 95 °C for 3 s and amplification and annealing at 60 °C for 20 s. A standard curve for each primer was used to calculate primer efficiency. mRNA expression levels of pro-inflammatory cytokines *IL-1a, IL-1b, IL-1r1, IL-6* and clock genes *Clock*, *Bmal1*, *Per1*, *Per2*, *Cry1*, *Cry2* and *Rev-Erba,* were normalized to housekeeping genes using the Pfaffl method [23] to obtain relative expression levels. *Gapdh* and *Rn18s* or ß-*Actin*, *Gapdh* and *Rn18s* were used as housekeeping genes for the relative expression levels of pro-inflammatory cytokines and clock genes, respectively. The quality of the PCR amplification product was confirmed by melting curves and agarose gel electrophoreses. The gene accession number was obtained from the NCBI database (Gene database/GenBanke) (https://www.ncbi.nlm.nih.gov/gene (accessed on 16 December 2022)). Amplicon size for the target genes was detected using Primer-BLAST (https://www.ncbi.nlm.nih.gov/tools/primer-blast/index.cg (accessed on 16 December 2022)). 

### 2.4. Immunohistochemistry

Mice from each group were sacrificed 48 h after irradiation/handling at four different time points: ZT02, ZT08, ZT14 and ZT20 via i.p. injection of ketamine/xylazine (100/10 mg/kg) then transcardially perfused with 0.9% NaCl followed by 4% formalin.

The brains were removed from the skull and post-fixed in 4% formalin for 24 h. Brains were cryoprotected in 20% sucrose, followed by 30% sucrose. Brains were cut on a Leica CM3050 cryostat (Leica Biosystems, Nussloch, Germany) into 30 µm thick coronal sections along the rostro-caudal extent of the hippocampus. Primary and secondary antibodies used for immunohistochemistry are listed in Table 2. Negative control staining was performed to test the specific binding of the antibody.

For chromogenic immunohistochemistry, sections were washed in phosphate-buffered saline (PBS) and incubated with 0.6% H_2_O_2_ for 30 min at room temperature (RT). To prevent non-specific staining, sections were pre-incubated in a blocking buffer of 5% normal goat serum in PBS-Triton 0.2% (PBST) for one hour at RT. Then, sections were incubated overnight at 4 °C with antibodies against markers for neuronal activity (c-FOS) or proliferation (Ki67). The sections were then washed in PBST and incubated with a biotin-conjugated secondary antibody for one hour at RT. The sections were then rinsed with PBST and then treated with the Vectastain Elite ABC kit (1:200, Cat # PK-6100, Vector Laboratories) for one hour at RT. This was followed by rinsing with PBST and then by incubation in 0.05% 3,3′-diaminobenzidine (DAB, Cat # 190774-58-4, Sigma Aldrich, St. Louis, MI, USA) for 5 min at RT. Sections were finally washed with PBS and coverslipped with DePeX (Serva Electrophoresis, Heidelberg, Germany).

For immunofluorescence, sections were washed and incubated with a blocking buffer for one hour. The sections were incubated overnight at 4 °C with antibodies against markers for microglial cells IBA1, astrocytes GFAP or the oxidative stress 8-hydroxy-2′-deoxyguanosine (8-OHdG) [24], and then were washed in PBST at RT. This was followed by incubation with the secondary antibodies conjugated with Alexa-fluorophores for one hour at RT. After washing, sections were mounted on slides and coverslipped with the anti-bleaching mounting medium Dapi-Fluoromount that included a marker for cell nuclei (Cat # 0100-20, Southern Biotech, Birmingham, AL, USA).

### 2.5. Image Analysis 

Prior to image acquisition, the experimental condition was obscured to the investigator. Images were acquired using a BZ-900E microscope (Keyence, Osaka, Japan). The microscope settings were kept constant for all images of one staining. c-FOS immunoreactive (+) and Ki67+ cells were recorded in the bright field mode with 40X objective. Immunoreactive cells were counted in a delineated area of the hippocampal dentate gyrus (DG) using BZ-II analyzer software (Keyence, Osaka, Japan); the mean cell density was calculated and displayed as number of cells/mm^3^. Immunofluorescent signals (IR) for IBA1, GFAP and 8-OHdG were recorded using 20X objectives and respective filters of KEYENCE BZ-900E fluorescent microscope. The mean intensity in the hippocampus was quantitatively analyzed above the background intensity and expressed as arbitrary units (A.U.) using Image J software (http://rsbweb,nih.gov/ij (accessed on 16 December 2022)) as previously described [25].

### 2.6. Western Blot

The mice were sacrificed 48 h after irradiation/handling at four different time points: ZT02, ZT08, ZT14 and ZT20 and the hippocampus was dissected and homogenized in RIPA buffer containing HALT proteinase and phosphatase inhibitor (Cat # 78442, Thermo Scientific, Waltham, MA, USA) using a PRECELLYS^®^ Evolution tissue homogenizer (Bertin Instruments, Montigny-le-Bretonneux). A BCA kit (Thermo Scientific, Waltham, MA, USA) was used to determine the protein concentration. Gel electrophoresis of independent samples and immunoblotting were performed using Novex XCell Sure Lock system and PVDF membranes as described [26]. Membranes of each group were divided at 50kDa into 2 parts: the lower part (contains proteins less than 50 kDa) was incubated with rabbit polyclonal anti-p-ERK (1:2500, Cell Signaling Technology, Danvers, MA, USA) and the upper part (contains proteins greater than 50 kDa) was incubated with rabbit polyclonal anti-ß-tubulin (1:10,000, Cat # ab76287, Abcam, Cambridge, UK) over night at 4 °C. After washing, the membranes were incubated with secondary HRP-conjugated goat anti-rabbit IgG (1:40,000, Cat # 81-1620, Thermo Scientific, Waltham, MA, USA). The respective bands were visualized using Immobilon Western ECL HRP Substrate (Cat # WBKLS0500, Merk-Millipore, Burlington, MA, USA) via Chemi Only Gel Documentation System (VWR). Intensity of p-ERK bands (44 and 42 kDa) was measured via ImageJ soft (Gels tool) and normalized against ß-tubulin bands (55 kDa). 

### 2.7. Statistical Analysis

GraphPad Prism 8 software (GraphPad Software, Inc., San Diego, CA, USA) was used for statistical analysis. Anderson–Darling, Kolmogorov–Smirnov and D’Agostino and Pearson tests were used to test the normality of the data. When the data were normally distributed, multiple *t*-tests were used to analyze significant differences between two groups at a specific time point, while the comparison between different time points within one group was performed using ordinary one-way analysis of variance (ANOVA) followed by Tukey’s post-hoc tests. When the data violated the normal distribution, the non-parametric Mann–Whitney-U test was used to compare two groups at a specific time point and Kruskal–Wallis followed by Dunn’s multiple comparisons test were applied to detect differences among multiple time points within one group. Data are expressed as mean ± standard error of the mean (SEM). The results were regarded as statistically significant if *p* < 0.05.

## 3. Results

### 3.1. Effect of HCC and Irradiation on the Expression Levels of Genes Encoding for Pro-Inflammatory Cytokines 

**Figure 1:**
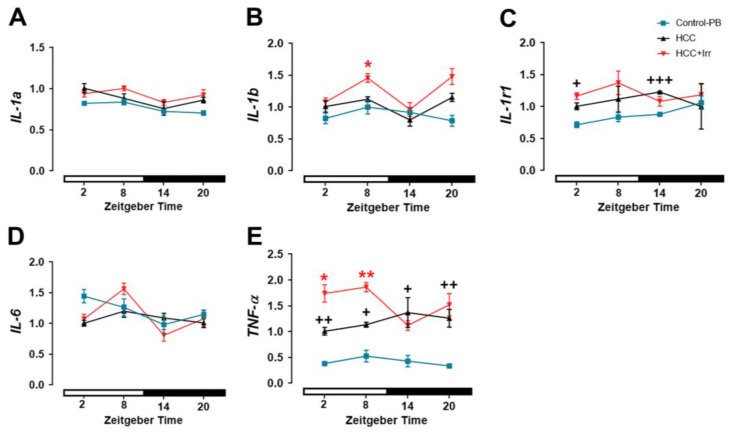
Effect of hepatocellular carcinoma (HCC) and radiotherapy on the relative expression of genes encoding for pro-inflammatory cytokines in the mouse hippocampus. (**A**) Relative expression of *IL-1a* mRNA. (**B**) Relative expression of *IL-1b* mRNA. (**C**) Relative expression of *IL-1r1* mRNA. (**D**) Relative expression of *IL-6* mRNA. (**E**) Relative expression of *TNF-α* mRNA. Black crosses indicate differences between HCC mice and control-PB. Red asterisks indicate differences between HCC and HCC+Irr. *, +: *p* < 0.05, **, ++: *p* < 0.01, +++: *p* < 0.001. White and black bars indicate the day/rest phase and night/activity phase, respectively. *n* = 12 mice per group, 3 mice at each time point. Control-PB: control mice given phenobarbital (PB) in drinking water. HCC: HCC-bearing mice. HCC+Irr: HCC-bearing irradiated mice. Interleukin (*IL*). Interleukin-1 receptor (*IL-1r1).* Tumor necrosis factor alpha (*TNF-α*).

As an indication of inflammation, the expression of genes encoding pro-inflammatory cytokines was investigated. We found that untreated mice showed peaks in the hippocampal expression of *IL-1a*, *IL-1b* and *TNF-α* during the light/inactive phase, which were blunted in control-PB mice (Supplementary Figure S1A,B,E). HCC-bearing mice showed a statistically significant higher hippocampal expression level of *IL-1r1* at ZT02 (*p* = 0.04) and ZT14 (*p* = 0.002) and of *TNF-α* at all-time points (*p* = 0.006 at ZT02, *p* = 0.01 at ZT08; *p* = 0.006 at ZT14, and *p* = 0.03 at ZT20) than control-PB mice (Figure 1). Irradiation during the dark/active phase did not result in significant changes in the hippocampal expression of pro-inflammatory cytokines (Figure 1A–E). In contrast, irradiation during the light/rest phase induced a significant increase in hippocampal *IL-1b* and *TNF-α* expression as compared to non-irradiated HCC-bearing mice (Figure 1B,E). Importantly, HCC or irradiation did not significantly affect the expression of pro-inflammatory cytokines genes in the liver (Supplementary Figure S2). 

### 3.2. Effect of HCC and Irradiation on Glial Cell Activation and Oxidative Stress Levels 

**Figure 2:**
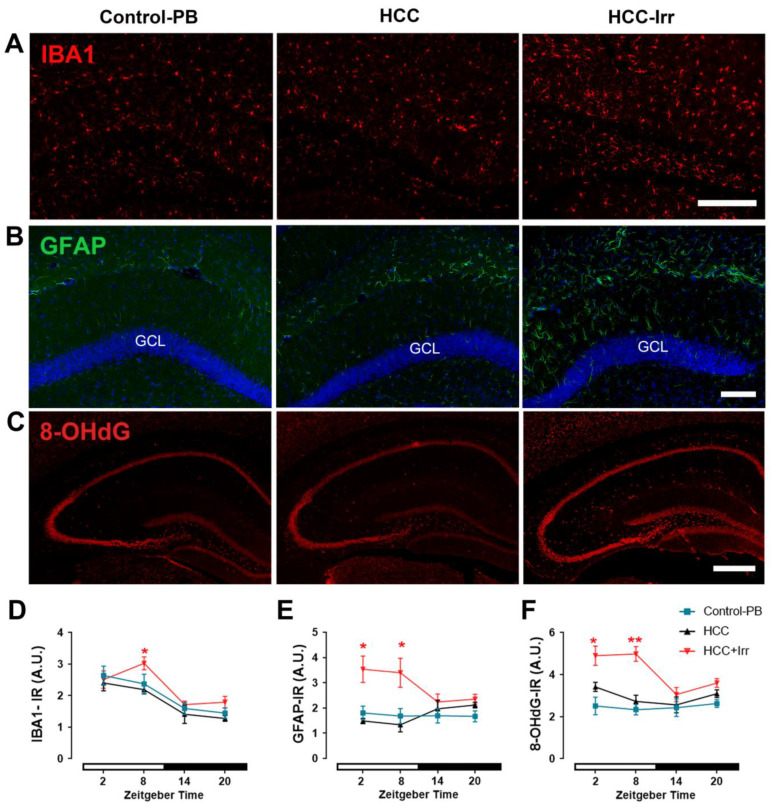
Effect of HCC and radiotherapy on glial activation and oxidative stress in the mouse hippocampus. (**A**) Representative photomicrographs of mouse hippocampal sections showing the immunoreaction (IR) of the microglial marker IBA1 (red) at ZT08 in control-PB, HCC and HCC+Irr mice. (**B**) Representative photomicrographs of mouse hippocampal sections showing IR of astrocytic marker GFAP (green) and nuclear staining DAPI (blue) at ZT08 in control-PB, HCC and HCC+Irr mice. (**C**) Representative photomicrographs of mouse hippocampal sections show IR of oxidative stress marker 8-OHdG (red) at ZT08 in control-PB, HCC and HCC+Irr mice. (**D**) Quantification of microglial activation assessed by IBA1-IR. (**E**) Quantification of astrocytic activation by GFAP-IR. (**F**) Quantification of oxidative stress by 8-OHDG-IR. Red asterisks indicate differences between HCC and HCC+Irr. *: *p* < 0.05, **: *p* < 0.01. White and black bars indicate the day/rest phase and night/activity phase, respectively. *n* = 12 mice per group, 3 mice at each time point, 2 sides per animal. Scale bar = 100 µm in (**A**,**B**); = 200 µm in (**C**). Control-PB: control mice given phenobarbital (PB) in drinking water. HCC: HCC-bearing mice. HCC+Irr: HCC-bearing irradiated mice. Glial fibrillary acidic protein (GFAP). 8-hydroxydeoxyguanosine (8-OHdG). Granular cell layer (GCL).

In the hippocampus of untreated mice, the marker for microglial activation, IBA1-IR, was constantly low; however, upon PB treatment, a peak during the light phase appeared (Supplementary Figure S3A). HCC had no additional effect on IBA1-IR; however, radiotherapy applied during the light phase (ZT08) resulted in a significant increase in IBA1-IR (*p* = 0.03) as compared to non-irradiated HCC-bearing mice (Figure 2A,D). PB administration (Supplementary Figure S3B) or HCC (Figure 2B,E) had no significant effect on GFAP-IR as a marker for astrocyte activation in the hippocampus. However, radiotherapy during the light phase resulted in a significant increase in GFAP-IR expression while radiotherapy applied during the dark phase had no effect on GFAP-IR in the hippocampus (Figure 2B,E). 

The marker for oxidative stress, 8-OHdG-IR, was not affected by PB treatment (Supplementary Figure S3C) or HCC (Figure 2C,F). However, irradiation during the light phase resulted in a significant increase in 8-OHdG-IR as compared to non-irradiated HCC-bearing mice, whereas the administration of radiotherapy during the dark phase had no effect (Figure 2C,F). 

### 3.3. Effect of HCC and Irradiation on Neuronal Activity and Neural Progenitor Cell (NPC) Proliferation 

**Figure 3:**
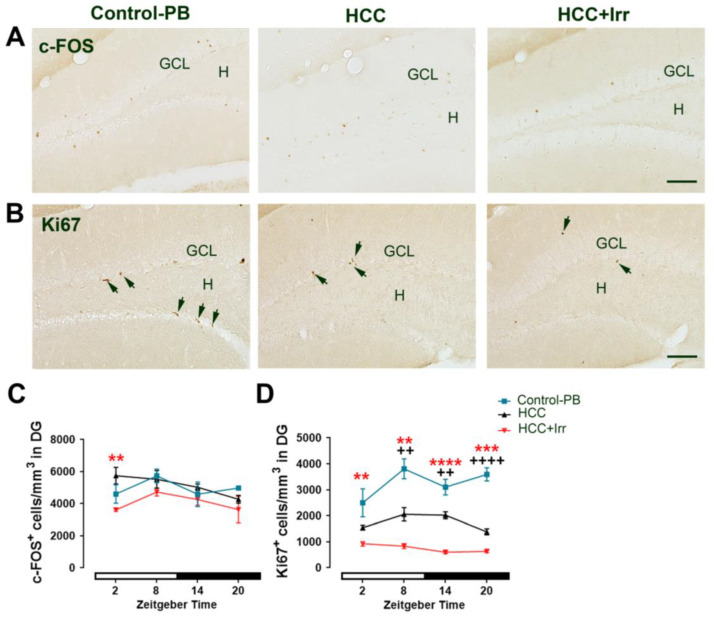
Effect of HCC and radiotherapy on neuronal activity and adult neurogenesis in the mouse hippocampus. (**A**) Representative photomicrographs of mouse hippocampal sections showing c-FOS+ neurons at ZT02 in control-PB, HCC-bearing mice and irradiated mice. (**B**) Representative photomicrographs of mouse hippocampal sections showing Ki67+ proliferating cells (black arrows) at ZT08 in control-PB, HCC-bearing mice and irradiated mice. (**C**) Quantification of cell density of c-FOS+ neurons/mm^2^ in dentate gyrus (DG). (**D**) Quantification of cell density of Ki67+ proliferating cells/mm^2^ in subgranular zone of the DG. Black crosses indicate differences between HCC mice and control-PB. Red asterisks indicate differences between HCC and HCC+Irr. **, ++: *p* < 0.01, *** *p* < 0.001, ****, ++++:: *p* < 0.0001. White and black bars indicate the day/rest phase and night/activity phase, respectively. *n* = 12 mice per group, 3 mice at each time point, 2 sides per animal. GCL: granule cell layer, H: hilus. Scale bar = 100 µm. Control-PB: control mice given phenobarbital (PB) in drinking water. HCC: HCC-bearing mice. HCC+Irr: HCC-bearing irradiated mice. Granular cell layer (GCL). Hilus (H).

In the granule cell layer (GCL) of the hippocampal dentate gyrus (DG), the number of c-FOS+ cells, a marker of neuronal activity, was not significantly different between untreated and PB-treated mice (Supplementary Figure S3D). HCC had no significant effect on the number of c-FOS+ cells as compared to PB-treated controls. However, irradiation at the early light phase (ZT02) resulted in a significant reduction in the number of c-FOS+ cells (*p* = 0.002), while irradiation at any other time point had no significant effect (Figure 3A,C). 

The number of the proliferating Ki67+ cells in the neurogenic niche within the DG was not different between untreated and PB-treated mice (Supplementary Figure S3E). HCC resulted in a significant reduction in Ki67+ cells as compared to PB-treated mice. Irradiation at all investigated time points resulted in a further significant reduction in the number of Ki67+ cells (Figure 3B,D)

### 3.4. Effect of HCC and Irradiation on Expression Levels of Clock Genes in the Hippocampus

**Figure 4:**
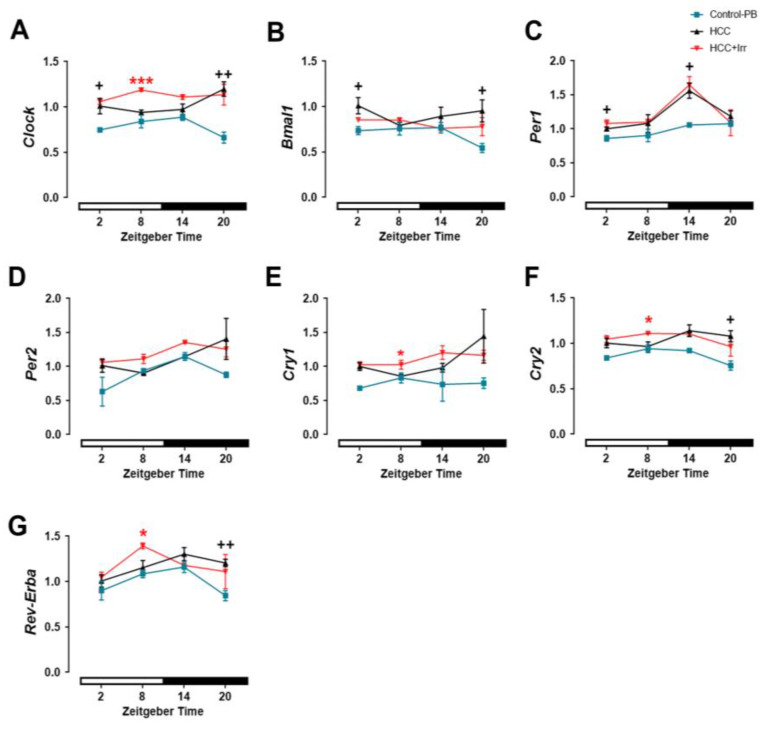
Effect of HCC and radiotherapy on clock gene expression in the mouse hippocampus. (**A**) Relative expression of *Clock* mRNA. (**B**) Relative expression of *Bmal1* mRNA. (**C**) Relative expression of *Per1* mRNA. (**D**) Relative expression of *Per2* mRNA. (**E**) Relative expression of *Cry1* mRNA. (**F**) Relative expression of *Cry2* mRNA. (**G**) Relative expression of *Rev-Erba* mRNA. Black crosses indicate differences between HCC mice and control-PB. Red asterisks indicate differences between HCC and HCC+Irr. *, +: *p* < 0.05, ++: *p* < 0.01, ***: *p* < 0.001. White and black bars indicate the day/rest phase and night/activity phase, respectively. *n* = 12 mice per group, 3 mice at each time point. Control-PB: control mice given phenobarbital (PB) in drinking water. HCC: HCC-bearing mice. HCC+Irr: HCC-bearing irradiated mice. Brain Muscle Arnt-like protein-1 (*Bmal1*). Period genes (*Per1, 2*). Cryptochrome genes (*Cry1, 2*).

In the hippocampus of untreated mice, relative *Clock*, *Bmal1*, *Per1*, *Cry1* and *Cry2* mRNA expression levels showed a time-of-day-dependent variation with a peak at ZT02 while *Per2* and *Rev-Erba* did not show a time-of-day-dependent variation (Supplementary Figure S4). The peaks of relative *Clock*, *Bmal1*, *Per1*, *Cry1* and *Cry2* mRNA expression were blunted in PB-treated mice (Supplementary Figure S4). However, compared to control-PB, HCC resulted in a significant upregulation of most clock genes in the hippocampus including *Clock* and *Bmal1* (at ZT02 and ZT20, Figure 4A, B), *Per1* (at ZT02 and ZT14, Figure 4C), *Cry2* (at ZT20, Figure 4F) as well as *Rev-Erba* (at ZT20, Figure 4G) mRNA expression. Only *Per2* and *Cry1* mRNA expressions were not significantly different between control-PB and HCC (Figure 4D,E). Irradiation at ZT02, ZT14 or ZT20 did not induce a significant change in the relative expression levels of any clock gene, whereas radiotherapy applied at ZT08 resulted in a significant increase in *Clock* (Figure 4A), *Cry1* (Figure 4E), *Cry2* (Figure 4F) and *Rev-Erba* (Figure 4G) mRNA levels. 

### 3.5. Effect of HCC and Irradiation on the Expression Levels of p-ERK in the Hippocampus

**Figure 5:**
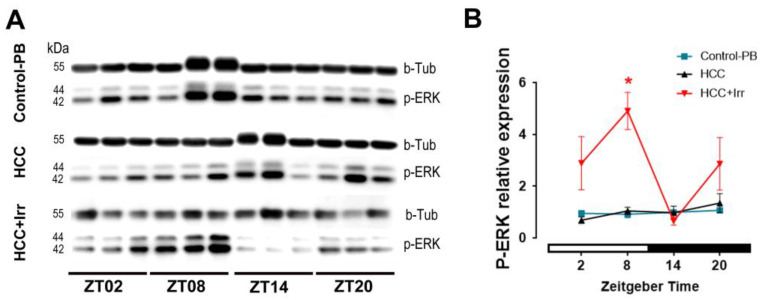
Effect of HCC and irradiation on levels of p-ERK in the hippocampus. (**A**) Immunoreactive bands and (**B**) quantification of p-ERK levels normalized to the housekeeper b-Tubulin in the hippocampus of control-PB, HCC-bearing and HCC+Irr mice. *: *p* < 0.05. White and black bars indicate the day/rest phase and night/activity phase, respectively. *n* = 12 mice per group, 3 mice at each time point. Control-PB: control mice given phenobarbital (PB) in drinking water. HCC: HCC-bearing mice. HCC+Irr: HCC-bearing irradiated mice. Phosphorylated extracellular signal-regulated kinase (p-ERK). b-Tubulin (b-Tub). Kilo Dalton (kDa). Zeitgeber time (ZT).

The relative protein levels of p-ERK in the hippocampus were not significantly different between PB-control mice and HCC-mice. Irradiation at the late light/inactive phase (ZT08) led to a significant increase in the protein levels of p-ERK compared to the HCC-mice (*p* = 0.02) (Figure 5). Irradiation at any other time point had no significant effect. 

## 4. Discussion

Here, we have shown that HCC resulted in an increased expression of pro-inflammatory cytokines and decreased proliferation and changes in clock gene expression in the hippocampus. Irradiation, in particular when applied in light/rest phase, enhanced all of these effects and also caused the activation of glia, oxidative stress and increased p-ERK protein levels while cFos-IR was decreased.

The hippocampal-dependent cognitive functions such as memory formation and extinction are largely time-of-day-dependent and are influenced by the circadian system [27]. In the hippocampus of untreated mice, the expression of clock genes except *Per2* and *Rev-Erba* showed a peak during the early light/inactive phase in agreement with previously reported findings [28,29]. Interestingly, expression of pro-inflammatory cytokines including *IL-1a*, *IL-1b* and *TNF-α* showed the same peak, suggesting an interaction of the molecular clockwork and the innate immune system in the hippocampus. There is increasing evidence of an interaction between the molecular clockwork and neuroinflammation [30,31]. In addition, studies using knock-out mice showed an interaction of *Bmal1* [32] and *Rev-Erba* [33] with the innate immune system of the brain. Importantly, in mice chronically treated with PB, which like other barbiturates works by increasing the activity of the inhibitory neurotransmitter GABA, these rhythms were blunted, suggesting that GABA affects the molecular clockwork and the innate immune system in the hippocampus. Similarly, chronic treatment with PB resulted in a reduction and less stable rhythm of spontaneous locomotor activity and affected the rhythms of serum corticosterone levels as well as SCN neuronal activity [17]. Surprisingly, upon PB treatment, a peak in IBA1-IR during the light/inactive phase appeared. This is consistent with an exaggerated reaction to immune stimulation of the hippocampal microglia isolated during the light phase [34,35,36,37]. 

In mice with HCC, the expression of pro-inflammatory cytokines and clock gene expression was increased in the hippocampus, indicative of a liver–brain crosstalk. Similarly, a recent study showed that circadian disruption enhanced breast cancer-mediated inflammation in the brain [37]. Moreover, disrupted circadian rhythms in the microglia may induce cytokine expression [34]. It has been suggested that signaling pathways between diseased liver and the brain are mediated by released cytokines that reach the brain through: (1) the neuronal route via the activation of vagal afferents to the brain or (2) the humoral route via the blood circulation to areas devoid of the blood–brain barrier (e.g., circumventricular organs) [38] leading to microglial activation and alteration in the neurotransmission system [9]. In addition, systemic inflammation [39], chronic liver diseases and carcinogenesis induce changes in systemic immunity together with monocyte adhesion along cerebral endothelial cells that are mediated by TNFα signaling and are also tightly associated with microglial activation and upregulation of several pro-inflammatory cytokines including IL-1a, IL-1b and IL-6 [7,40,41]. Moreover, liver pathology results in changes to astrocyte function and morphology such as the upregulation of GFAP, known as reactive astrogliosis, which is involved in the restoration of homeostasis and the protection of brain tissue [42], presumably mediated by circulating lipopolysaccharides and ammonia, as well as cytokines [43]. Furthermore, liver growth factor, a hepatic mitogen that remarkably increases in response to liver disorders promotes microglia activation and reactive astrogliosis [44]. However, HCC had a significant impact on proliferation in the hippocampal DG, which is one of the main neurogenic niches. Adult neurogenesis in the hippocampus is linked to learning and memory [45]; thus, poor memory in cancer [9] might be, at least partially, due to affected proliferation of neural stem/progenitor cells (NPCs). Proliferation of NPCs is controlled by various extrinsic and intrinsic regulators, including clock genes, neurotrophic and growth factors [46]. Bone morphogenetic protein 9 (BMP9), which is upregulated in HCC [47], is also known to control cell proliferation [48]. Moreover, biliary cholangitis, which is associated with liver diseases, results in reduced hippocampus volume and memory decline [49]. Moreover, pro-inflammatory cytokines such as IL-6 [50], IL-1b [51], and TNF-α [39] are known to affect adult neurogenesis.

Radiotherapy is known to induce pro-inflammatory responses and microglial activation in the hippocampus [52], astrogliosis [53], oxidative stress and generation of free radicals leading to tissue damage [54,55] as well as cognitive impairments due to inhibited adult neurogenesis [45,53,56]. Our previous data showed that the implication of chronotherapy for the treatment of HCC by irradiation might enhance the antimitotic effect on the tumor tissue and reduce the side effects [16,17]. Here, we showed that irradiation, particularly applied in the light/inactive phase resulted in the increased expression of clock genes *Clock*, *Cry1*, *Cry2*, *Rev-Erba* and the pro-inflammatory cytokines *IL-1b*, *TNF-α*, oxidative stress and the activation of microglia and astrocytes. Clock genes are important players in microglial activation and pro-inflammatory responses [36,57]. Interestingly, there was no time-of-day-dependent effect of irradiation on the expression of pro-inflammatory cytokines in the liver. Thus, the pro-inflammatory response to irradiation in the light phase in the hippocampus might be a consequence of the exaggerated reaction of the hippocampal innate immune system (see above) rather than a systemic effect. In line with this argument, the irradiation during the early light/inactive phase had the lowest impact on plasma glucocorticoid levels [17]. In contrast, the inhibition of proliferation in the hippocampus was not dependent on the timing of irradiation, suggesting that the cell cycle of NPCs does not have a comparable time window of vulnerability to mature glial cells. Remarkably, irradiation in the early light/inactive phase also had the highest impact on the marker for neuronal activity, c-FOS, in the hippocampus. Thus, it is tempting to speculate that during the light phase, a higher vulnerability of hippocampal glia to irradiation does not only result in changes to molecular clockwork and innate immune function but also leads to changes in neuronal activity. This might involve microRNAs (miR-132/miR-212, miR-134) which affect hippocampal c-FOS expression upon irradiation [53] and are known to modulate the expression of pro-inflammatory cytokines [58]. The extracellular signal-regulated kinase 1/2 (ERK1/2) signaling pathway is involved in multiple essential brain functions including synaptic plasticity, neuronal repair and neuroinflammation [59]. It has been shown that irradiation enhances ROS generation, which results in the activation of the ERK1/2 pathway. Activated p-ERK1/2 subsequently leads to the phosphorylation of the transcription factor c-Jun, which regulates genes encoding for pro-inflammatory cytokines, including TNF-α and IL-1β [60]. MAPK/ERK signaling pathway induces microglial NADPH activation that contributes to neuroinflammatory reactions [61]. Additionally, oxidative stress and the activated ERK pathway stimulate PARP-1, which mediates microglia and astroglial responses to neuroinflammation induced by irradiation [62]. The MAPK/ERK pathway modulates the circadian genes through the direct interaction and phosphorylation of CLOCK, BMAL1, CRY1 and CRY2 or via CREB phosphorylation, which directly regulates the transcription of the per1 and per2 genes [63]. Irradiation during the light phase induced the most pronounced p-ERK levels in the hippocampus, consistent with activated glial cells and there was an increase in pro-inflammatory cytokines and an alteration of circadian genes when irradiation was applied at this time point. Thus, we assume that the time-of-day-dependent changes induced by radiotherapy during the light/inactive phase were mediated via the ERK1/2 pathway. However, the downstream targets remain to be elucidated. It should be addressed in future studies whether the here observed changes in the gene expression of pro-inflammatory cytokines and clock genes are associated with the respective changes in protein levels as post-translational modifications cannot be excluded. Furthermore, future studies should address the effects of HCC and time-of-day-dependent radiotherapy on hippocampal functions such as spatial memory and learning. 

## 5. Conclusions

In conclusion, our study supports the hypothesis of an interaction of the molecular clockwork and the innate immune system and that pro-inflammatory cytokines are key players in liver–brain crosstalk in the context of liver cancer and thus, might help to refine therapeutic targets for the amelioration of central symptoms. Most importantly, we showed that radiotherapy induced the most profound effects on the expression of pro-inflammatory cytokines and clock genes, glial activation, oxidative stress and neuronal activity in the hippocampus when applied during the light/inactive phase, presumably via an ERK pathway-related mechanism. Thus, radiotherapy application during the (late) active phase not only has the highest efficacy on tumor tissue and the least side effects on liver tissue as we showed earlier but also the least side effects on the hippocampus. However, translational studies are needed to prove this hypothesis in human patients.

## Figures and Tables

**Table 1:** List of primers used for real time qPCR.

Primer	Forward	Reverse	Amplicon Size	GenBank Accession Number
*IL-1a*	CTACAGTTCTGCCATTGACCA′	ACTCAGCCGTCTCTTCTTCAG	211 bp	NM_010554.4
*IL-1b*	AGCCTGTGTTTTCCTCCTTGC	TCAGTGCGGGCTATGACCAA	171 bp	NM_008361.4
*IL-1r1*	ACCAAACCTGTGCAGTCCCT	TGGCCACCAAGTCCTGTTCT	72 bp	NM_008362.3
*IL-6*	AGTCCTTCCTACCCCAATTTCCA	TGGTCTTGGTCCTTAGCCACT	80 bp	NM_001314054.1
*TNF-α*	CCCTCACACTCAGATCATCTTCT	GCTACGACGTGGGCTACAG	61 bp	NM_001278601.1
*Clock*	CAC CGA CAA AGA TCC CTA CTG AT	TGA GAC ATC GCT GGC TGT GT	151 bp	NM_001305222.1
*Bmal1*	GTA GAT CAG AGG GCG ACA GC	CCT GTG ACA TTC TGC GAG GT	114 bp	NM_001243048.2
*Per1*	TGG CTC AAG TGG CAA TGA GTC′	GGC TCG AGC TGA CTG TTC ACT	247 bp	NM_001159367.2
*Per2*	CCAAACTGCTTGTTCCAGGC	ACCGGCCTGTAGGATCTTCT	153 bp	NM_011066.3
*Cry1*	CTT CTG TCT GAT GAC CAT GAT GA	CCC AGG CCT TTC TTT CCA A	151 bp	NM_007771.3
*Cry2*	AGG GCT GCC AAG TGC ATC AT	AGG AAG GGA CAG ATG CCA ATA G	151 bp	NM_009963.4
*Rev-erba*	GGT GCG CTT TGC ATC GTT	GGT TGT GCG GCT CAG GAA	64 bp	NM_145434.4
*Gapdh*	CAA CAG CAA CTC CCA CTC TTC	GGT CCA GGG TTT CTT ACT CCT T	164 bp	NM_001289726.2
*ß-Actin*	GGCTGTATTCCCCTCCATCG	CCAGTTGGTAACAATGCCATGT	154 bp	NM_007393.5

**Table 2:** List of antibodies used for immunohistochemistry and immunofluorescence.

Primary Antibody (Host, Clonality)	Manufacturer	Concentration
Anti-IBA1 (rabbit, polyclonal, cat # 019-19741)	Fujifilm WAKO (Osaka, Japan)	1:2000
Anti-GFAP (mouse, monoclonal, cat # 556330)	BD Biosciences (Eysins, Switzerland)	1:500
Anti-8OHdG (mouse, monoclonal, cat # AM03160PU)	Acris (San Diego, CA, USA)	1:2000
Anti-c-FOS (rabbit, monoclonal, cat # 2250)	Cell Signaling Technology (Danvers, MA, USA)	1:5000
Anti-Ki67 (rabbit, polyclonal, cat # ab16667)	DCS Immunoline (Hamburg, Germany)	1:500
Secondary antibody (host)	Manufacturer	Concentration
Anti-rabbit IgG Biotin (goat, cat # BA-1000)	Vector Laboratories (Burlingame, CA, USA)	1:500
Anti-rabbit IgG Alexa Fluor 568 (goat; cat # A-11036)	Molecular Probes (Eugene, OR, USA)	1:500
Anti-mouse IgG Alexa Fluor 568 (goat; cat # A-11031)	Molecular Probes (Eugene, OR, USA)	1:500
Anti-mouse IgG Alexa Fluor 488 (goat, cat # A-21042)	Molecular Probes (Eugene, OR, USA)	1:500

## Data Availability

The dataset supporting the conclusions of this article is available upon reasonable request to the corresponding author.

## Supplementary Materials

The following supporting information can be downloaded at: https://www.mdpi.com/article/10.3390/cells12010061/s1, Figure S1: Rhythmic expression levels of and effect of PB treatment on relative expression of genes encoding for pro-inflammatory cytokines in mouse hippocampus; Figure S2: Effect of hepatocellular carcinoma (HCC) and radiotherapy on relative expression of genes encoding for pro-inflammatory cytokines in mouse liver; Figure S3: Effect of PB treatment on glial activation, oxidative stress, neuronal activity and adult neurogenesis in mouse hippocampus; Figure S4: Rhythmic expression levels of and effect of PB treatment on relative expression of clock genes in mouse hippocampus.
