# Supplementary material for: Radiotherapy of the Hepatocellular Carcinoma in Mice Has a Time-Of-Day-Dependent Impact on the Mouse Hippocampus
Source: Cells. 2022 Dec 23;12(1):61. doi: 10.3390/cells12010061 (PMC9818790; doi:10.3390/cells12010061)
Supplement: Supplementary file 1 [file cells-12-00061-s001.zip › supplementary.pdf]

Supplementary Materials:

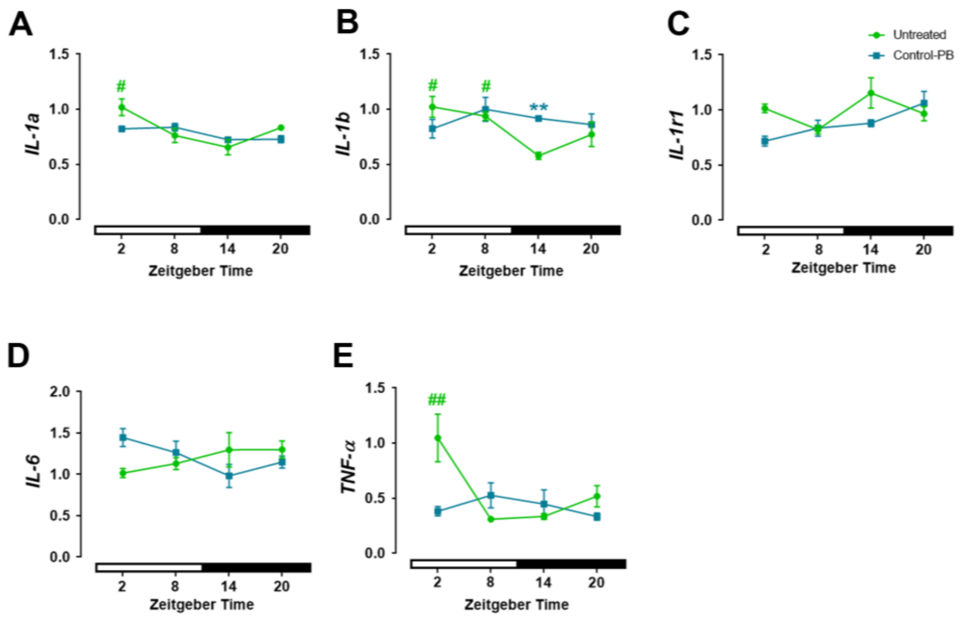

Figure S1. Rhythmic expression levels of and effect of PB treatment on relative expression of genes encoding for pro-inflammatory cytokines in mouse hippocampus. A) Relative expression of *IL-1a* mRNA. B) Relative expression of *IL-1b* mRNA. C) Relative expression of *IL-1r1* mRNA. D) Relative expression of *IL-6* mRNA. E) Relative expression of *TNF-α* mRNA. Green hashtag indicates differences between time point and ZT14 in the untreated group. #: $P < 0.05$, ##: $P < 0.01$. Blue asterisk indicates differences between untreated and control-PB (mice received phenobarbital in drinking water). **: $P < 0.01$. White and black bars indicate day and night, respectively. n = 12 mice per group, 3 mice at each time point.

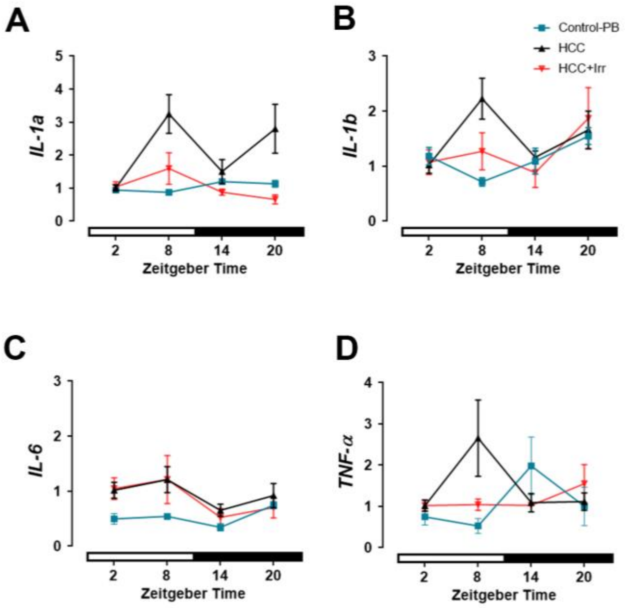

Figure S2. Effect of hepatocellular carcinoma (HCC) and radiotherapy on relative expression of genes encoding for pro-inflammatory cytokines in mouse liver. A) Relative expression of *IL-1a* mRNA. B) Relative expression of *IL-1b* mRNA. C) Relative expression of *IL-6* mRNA. D) Relative expression of *TNF-α* mRNA. HCC: HCC-bearing mice. HCC+Irr: HCC-bearing irradiated mice ~~received irradiation~~. White and black bars indicate day and night, respectively. n = 12 mice per group, 3 mice at each time point.

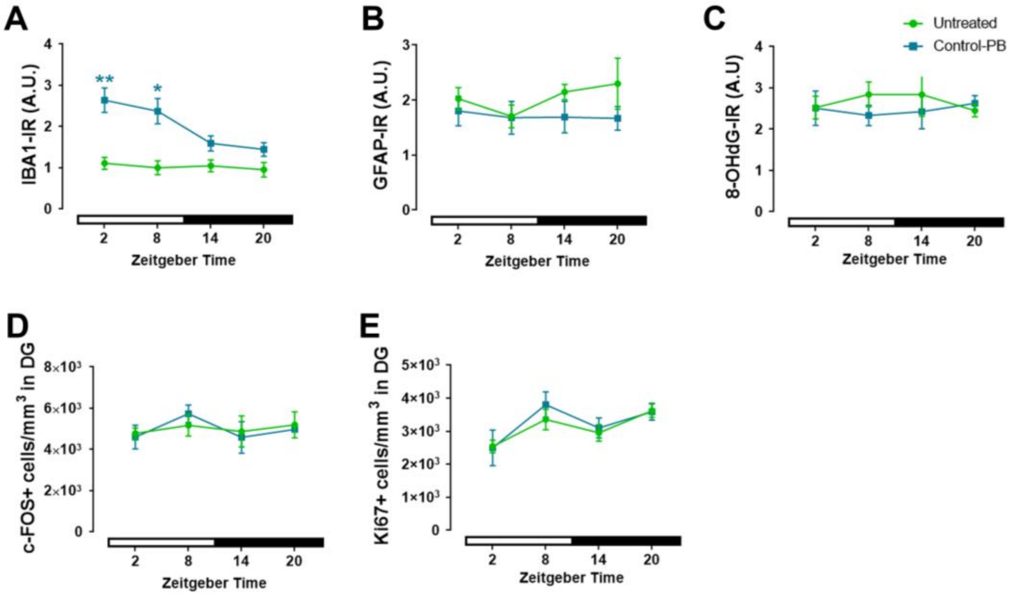

Figure S3. Effect of PB treatment on glial activation, oxidative stress, neuronal activity and adult neurogenesis in mouse hippocampus. A) Quantification of microglial activation assessed by IBA1 immunoreaction (IR). B) Quantification of astrocyte activation by GFAP-IR. C) Quantification

of oxidative stress marker 8-OHdG-IR. D) Quantification of number of c-FOS+ cells in dentate gyrus (DG). E) Quantification of number of KI67+ proliferating cells in DG. Blue asterisks indicate differences between untreated and control-PB.*: P < 0.05, **: P < 0.01. White and black bars indicate day and night, respectively. n = 12 mice per group, 3 mice at each time point, 2 sides per animal.

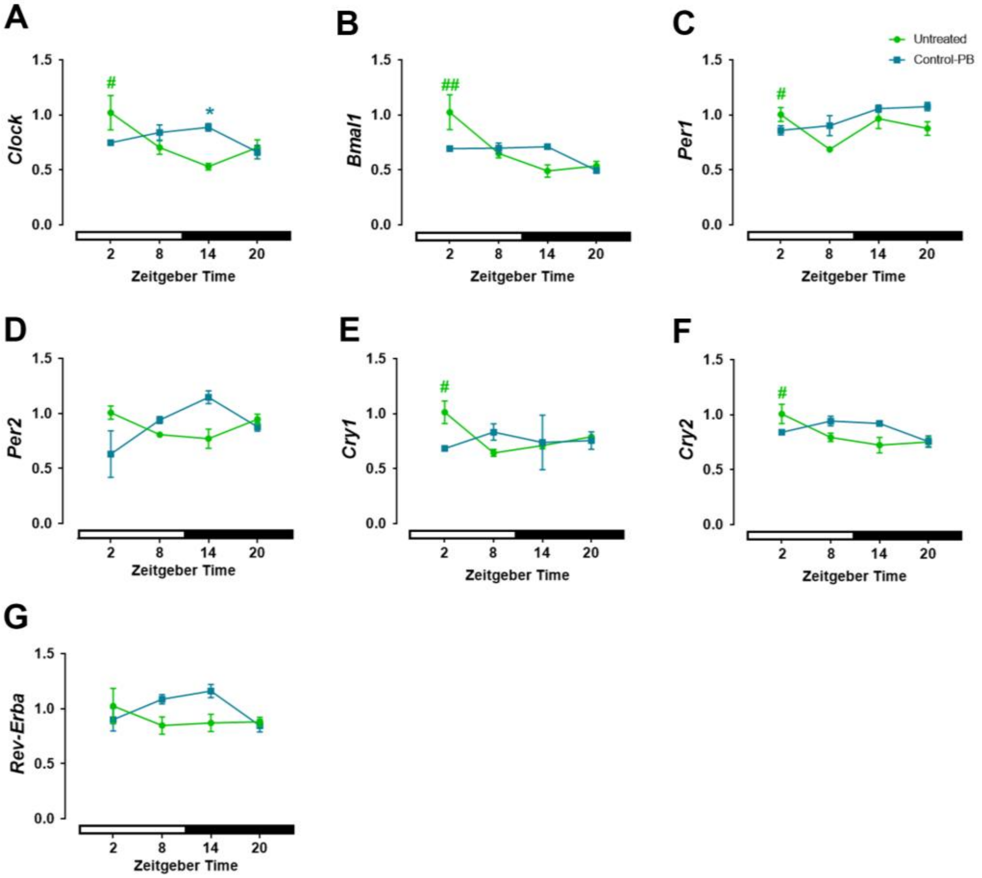

Figure S4. Rhythmic expression levels of and effect of PB treatment on relative expression of clock genes in mouse hippocampus. A) Relative expression of *Clock* mRNA. B) Relative expression of *Bmal1* mRNA. C) Relative expression of *Per1* mRNA. D) Relative expression of *Per2* mRNA. E) Relative expression of *Cry1* mRNA. F) Relative expression of *Cry2* mRNA. F) Relative expression of *Rev-Erba* mRNA. Green hashtag indicates differences between this time point and ZT14 in the untreated group. #: P < 0.05, ##: P < 0.01. Blue asterisk indicates differences between untreated and control-PB. *: P < 0.05. White and black bars indicate day and night, respectively. n = 12 mice per group, 3 mice at each time point.
